# Trends in Cervical Cancer Mortality in Brazilian Women who are Screened and Not Screened

**DOI:** 10.31557/APJCP.2020.21.1.55

**Published:** 2020

**Authors:** Adriana Cunha Vargas, Vargas Agnolo, Willian Augusto de Melo, Fernando Castilho Pelloso, Lander dos Santos, Maria Dalva de Barros Carvalho, Sandra Marisa Pelloso

**Affiliations:** 1 *Department of Health Sciences, *; 3 *Department of Medicine, Maringá State University, Maringa, Paraná, *; 2 *Department of Nursing, Paraná State University, UNESPAR Av. Gabriel Esperidião, S / N - Jd. Morumbi, Paranavaí, Brazil. *

**Keywords:** Cervical cancer, epidemiology, female, prevention and control

## Abstract

**Objective:**

This study aimed to analyze the trend in cervical cancer (ICD C53) mortality in Brazilian regions in women who are who are screened and not screened from 1996 to 2015.

**Methods:**

An epidemiological study, of time series of mortality from cervical cancer performed in 90,856 women under 24 years old (343 women), between 25 and 64 years old (32,703 women), and over 65 years old (10,909 women). The data from this research were collected from the DATASUS, from the SIM Health Surveillance Secretariat files, captured through TABNET selecting the resident population by gender and age group and ICD 10 C53 from 1996 to 2015.

**Results:**

Among women, 43.8% were white, and 76% had less than eight years of formal education. Polynomial regression showed an increasing trend in cervical cancer mortality in Brazil for women aged 15 - 24 years (p=0.01). Between 25 - 64 and 65 years or older it remained constant, but high (p=0.07; 0.99). The Northeast region pointed a growing trend in women aged 15 to 24 (p=0.01), 25 to 64 years (p=0.01) and 65 or older (p=0.001). The Northeast presented the highest average growth per year. In the Southeast, South and Midwest regions, decreasing trends were observed despite the high rates. The Joinpoint regression showed a 95% confidence interval, and that mortality from cervical cancer in the North region increased throughout the period analyzed. an increasing trend was observed from 1996 to 1998, whereas in the Midwest region, the trend remained stable throughout the period analyzed. The Federal District presented an upward trend from 1996 to 2015. In Brazil, an upward trend was observed throughout the whole period analyzed.

**Conclusions:**

Cervical cancer mortality in younger women is becoming more predominant, in addition to the high rate observed for women aged 65 or older.

## Introduction

Cervical cancer (CC) is the second most common form of cancer in women in less developed regions, with an estimated 570,000 new cases worldwide in 2018, corresponding to approximately 7.5% of all cancer in women (WHO, 2019; Ferlay et al., 2018). In that same year, approximately 311,000 women died from CC, with more than 85% of these deaths in low- and middle-income countries (WHO, 2019).

For Brazil in the biennium 2018-2019, there is an estimated occurrence of 420,000 new cases of cancer each year, excluding non-melanoma skin cancer, and 16,370 new cases of CC each year, with an estimated risk of 15.43 cases per 100,000 women, making it the third most common type of cancer (Inca, 2018). In the world, one million women currently live with CC, and many do not have access to health services for prevention and treatment (WHO, 2016).

In developed countries, there are programs that provide human papillomavirus (HPV) vaccination to girls and regular screening for women. The latter allows the identification of precancerous lesions at stages for which women can still be easily treated. Early treatment prevents up to 80% of CC cases. In developing countries, there is limited access to preventive measures. Thus, CC is often only identified at more advanced stages when symptoms have already developed. Because access to advanced surgical, radiotherapeutic or chemotherapeutic treatment is also limited, there are more CC-related deaths in those countries (WHO, 2019).

There are several studies on CC mortality in women aged 25-64 years, which is the age range defined for inclusion in screening programs in Brazil (Sousa, 2016; Madeiro, 2016). However, studies on CC mortality distribution using temporal trends for women not included in screening programs, assessing regional and state disparities in Brazil, are still scarce, although they are essential for understanding the disease.

One study shows that many women aged less than 25 years and more than 64 years do not participate in screening programs; there is little scientific evidence regarding incidence rates for women in these age groups (Lim et al., 2013). A study of women younger than 30 years found no association between CC screening and mortality, suggesting that CC is a rare disease in this age group (Vicus, 2014). Another study shows that the predominant screening pattern is opportunistic for women under the age of 25 and over the age of 64, i.e., women receive a Pap smear when they seek care for other reasons (Inca, 2016).

Thus, this study aimed to analyze the mortality trend from cervical cancer (ICD C53) in Brazil, regions and states of women who are included and not included in screening programs.

## Materials and Methods

This was an epidemiological study of time series of CC mortality data for women Brazilian women who are screened and not screened. The mortality data were extracted from electronic databases of the Department of Health (DATASUS) of the Brazilian public healthcare system (Unified Health System - SUS), the Mortality Information System (SIM) and the Brazilian Institute of Geography and Statistics (IBGE). Deaths from CC were included and classified as C53 according to the nomenclature of the International Classification of Diseases (ICD-10). 

The data collected covered the period from 1996 to 2015. This evaluation period was selected because 1996 was the year when the SIM began to record causes of death according to the International Classification of Diseases, Tenth Revision (ICD-10) guidelines.

The data were first collected for all age groups and then separated into three groups: women aged less than 24 years (343 women), between 25 and 64 years (32,703 women), and above 65 years (10,909 women). The second group was used for comparison purposes. This population was selected to analyze whether mortality rates are increasing in Brazilian regions and states as well as to assess whether rates are increasing among women not included in organized screening programs.

The variables included in this study were age, year of death, education level, marital status, place of residence and population. Mortality rates were calculated using deaths that occurred from 1996 to 2015 in Brazilian states and regions. These data were extracted from the DATASUS website (http://datasus.saude.gov.br/), from the SIM Health Surveillance Secretariat files, captured through TABNET selecting the resident population by gender and age group and ICD 10 C53 from 1996 to 2015. The SIM files were tabulated through TABWIN and were consolidated: Brazil by region and unit. The populations had as source the IBGE (Brazilian Institute of Geography and Statistics). The mortality rates were calculated as the number of deaths from cervical cancer (ICD C53) in women divided by the total female population in that year and place, which was obtained from demographic information from the 2000/2010 Census and estimates for the years of 1996 to 1999 and 2011 to 2015, multiplied by 100,000 For trend analysis, two regression models were used: polynomial and Joinpoint. The polynomial regression model used rates as dependent variables (x) and years as independent variable (y). The variable “year” was transformed into a variable centered on the year (x-2003), and the series was analyzed using a three-point moving average. These models were tested as linear (y = β_0_ + β_1_x_1_), quadratic (y = Β_0_ + β_1_x_1_ + β_2_x_2_) and cubic (y = β_0 _+ β_1_x_1_ + β_2_x_2_ + β3x3), considering as significant trends that obtained p <0.05.

The coefficient of determination (r^2^) was used to determine the model that best fit the data. When all the criteria were significant for more than one model and the coefficients of determination were similar, the simplest model was chosen. SPSS software version 20.1 was used to analyze the data.

The Joinpoint Regression Program, provided by the Surveillance Research Program of the USA National Cancer Institute, used year of death as the independent variable and rate as the dependent variable, according to the regions of Brazil. The models were fitted assuming different numbers of joinpoints, from zero (trend represented by a single line segment) to three, considering change points in the time evolution of the rates. As several tests were performed, Bonferroni correction was used to ensure that the overall probability of type I error was less than the specified significance level (α level, standard α = 0.05). Bonferroni correction was conservative because the actual overall significance level was generally lower than the nominal α level. The new correction procedure controlled the overall probabilities of having at least one false discovery (National Cancer Institute, 2018).

A map was constructed to show the temporal distribution of CC mortality rates, by mortality rate between Brazilian states and regions. The map was built using QGIS version 2.8.

The research followed the ethical procedures according to CNS Resolution n. 466/2012, however, public databases were used (http://datasus.saude.gov.br/).

## Results

In the years 1996 to 2015, 90,856 CC deaths were analyzed for Brazilian women aged 15 to > 80 years, of whom 43.8% were white, 76% had less than eight years of formal education, 59.6% had no husband or partner, and 74.4% were between 25 and 64 years of age (Table 1).

The polynomial regression analysis showed an increasing trend of CC mortality in Brazil among women aged 15 to 24 years, with a rate of 0.14 and an annual growth of 0.004, r^2^ = 0.46 and p = 0.01. The mortality rates for women aged 25-64 years and 65 years or older remained constant, but with high rates, 6.9% and 22.1%, respectively. The Northeast and Southeast regions showed an increasing mortality trend for women aged 15-24 years (p=0.01), 25-64 years (p=0.01) and 65 or more (p=0.001). In the Southeast region, the mortality rate had a decreasing trend, but increased during more recent years. The South region exhibited a mortality rate of 0.18. A similar result was observed in the Midwest region, where the mean mortality rate was 0.14.

The CC mortality trends in Brazil for women aged 25-64 years remained constant (6.9 p=0.07). In Brazilian, the North and Northeast regions showed an increasing trend, with a higher increase in the mortality rate in the North region (17.4). The Northeast region presented the highest mean annual growth. In the Southeast, South and Midwest regions, a decreasing trend was observed despite the high mortality rates. The decreasing trend in the South region had an annual mean of - 0.18, r^2^ = 0.77.

The mortality rate for women aged 65 years or older in Brazil was 22.01. The North region had the highest mortality rate for this age group, 40.3, which continually increased and remained the highest annual mean. The mortality rate in the Northeast region (23.11) continually increased; the annual mean was 0.71 r^2^= 0.76 p = <0.001. The lowest rate for this age group was obtained for the Southeast region (18.4), with a decreased annual growth of – 0.412 r^2^ = 0.80 p= 0.01. The South region also showed a decreasing trend, with a decrease of 0.33 r^2^ = 0.59 p= <0.01 per year. The Midwest region, although the mortality rate remained constant, had the second highest rate (30.7) among the regions, with an annual decrease of - 0.30 (Table 2).

**Figure 1:**
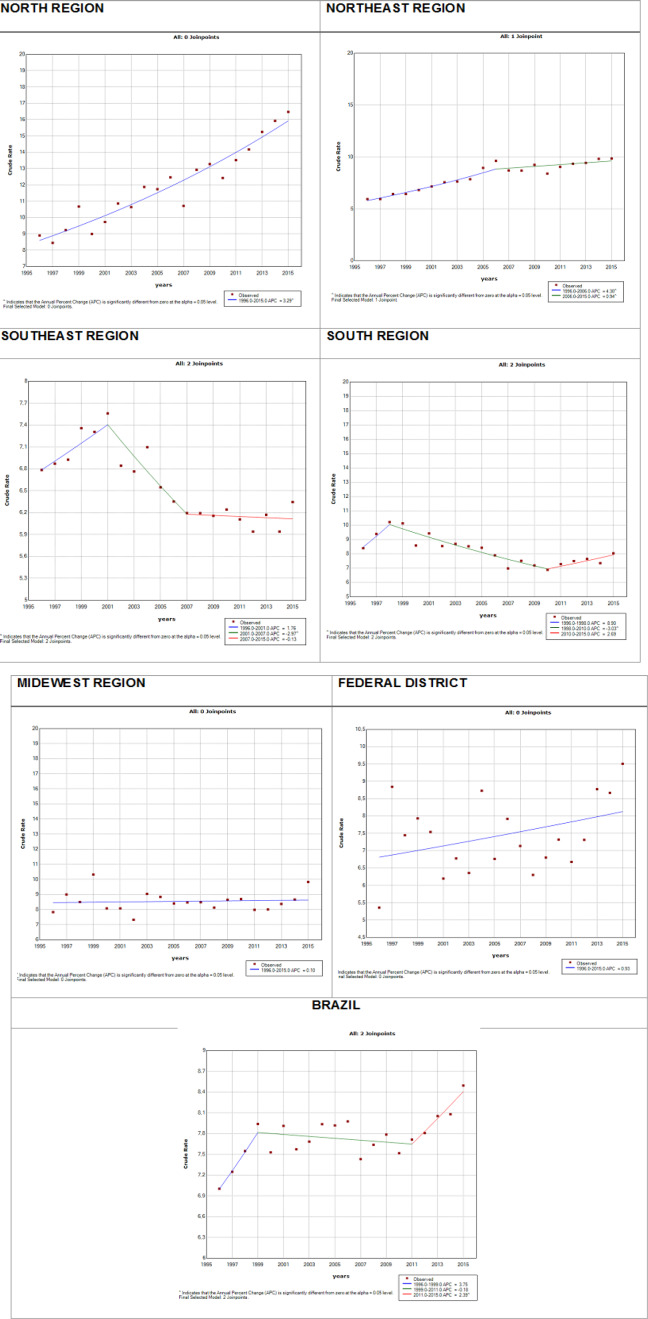
Trend Models of Mortality Rates from Cervical Cancer in Brazilian Women

The Joinpoint regression analysis showed that CC mortality in the Northern region increased throughout the period analyzed (annual percent change (APC) = 3.9^ from 1996 to 2015). In the Northeast region, an increasing trend was observed from 1996 to 2006 (APC = 4.30^), after which there was a slight decrease but still an upward trend (2006 to 2015, APC = 0.94^). The Southeast region exhibited trend changes during the studied period, with an initial increase followed by a strong decrease. The South region exhibited an upward trend for the 2-year period from 1996 to 1998, whereas from 1998 until 2010, the mortality rate in this region significantly declined, after which it increased again. In the Midwest region, the trend remained stable throughout the studied period. The mortality rate in the Federal District showed an upward trend from 1996 to 2015. In Brazil, an upward trend was observed throughout the whole period analyzed, with slight oscillations, and a strong increase in the last period (Figure 1).

The highest rates were found in the North region, indicating the increase in CC mortality in Brazil. The regions and states that previously experienced declines showed significant increases (Figure 3).

**Table 1:** Cervical Cancer Mortality Proportion in Brazilian Women who are Screened and not Screened from 1996 to 2015

Variables (Region)	North		Northeast		Southeast		South		Midwest		Brazil	
	%	n	%	n	%	n	%	n	%	n	%	n
Ethnicity												
White	3.8	1,515	14.7	5,865	46.1	18,385	28.6	11,398	6.7	2,678	43.8	39,841
Brown	20.3	6,426	42.8	13,574	25.5	8,074	2.6	826	8.8	2,802	34.9	31,702
Black	6.7	441	31.1	2,039	47.1	3,086	9.1	596	6.0	390	7.2	6,552
Yellow	6.5	34	27.1	141	53.6	279	7.9	41	5.0	26	0.6	521
Indigenous	42.6	143	17.9	60	6.8	23	9.5	32	23.2	78	0.4	336
Ignored	7.7	914	32.8	3,903	38.4	4,579	13.1	1,557	8.0	957	13.1	11,910
Total	10.4	9,473	28.2	25,582	37.9	34,426	15.9	14,450	7.6	6,931	100	90,862
Education level (years of study)												
<8	12.2	4,091	23.7	7,947	39.3	13,163	17.7	5,932	7.2	2,402	76.3	33,535
>8	14.6	1,522	19.9	2,073	40.3	4,198	17.0	1,774	8.2	855	23.7	10,422
Total	12.8	5,613	22.8	10,020	39.5	17,361	17.5	7,706	7.4	3257	100	43,957
Had a partner/husband												
With partner/husband	12.9	2,038	12.9	3,704	36.7	5,784	19.5	3,077	7.2	1,138	35.8	15,741
Without partner/husband	11.9	3,121	11.9	5,725	42.4	11,101	16.3	4,280	7.5	1,952	59.6	26,179
Other	22.3	454	22.3	591	23.4	476	17.1	349	8.2	167	4.6	2,037
Total	12.8	5,613	12.8	10,020	39.5	17,361	17.5	7,706	7.4	3,257	100	43,957
Age group (years)												
15 - 24	16.6	57	25.9	89	33.8	116	16.0	55	7.6	26	0.8	343
25 - 64	13.7	4,468	23.0	7,510	38.3	12,515	17.4	5,704	7.7	2,506	74.4	32,703
>65	10.0	1,087	22.2	2,421	43.3	4,729	17.8	1,947	6.6	725	24.8	10,909
Total	12.8	5,612	22.8	10,020	39.5	17,360	17.5	7,706	7.4	3,257	100	43,955

**Table 2:** Trends of Cancer of the Cervical Cancer of Brazilian Women who are Screened and Not Screened

Region	15 a 24 years				25-64 years				65 or more years			
	Model	R²	p	T*	Model	R²	p	T*	Model	R²	p	T*
Brazil	y=0.14+0.004x+0.0005x²	0.46	0.01	↑	y=6.9-0.06x+0.01x²+0.01x³	0.16	0.07	─	y=22.01-0.01x-0.02x²	0.01	0.99	─
North	y=0.26-0.001x+0.001x³	0.03	0.79	─	y=11.4+0.251x	0.81	<0.01	↑	y=40.3+1.57x	0.90	<0.01	↑
Northeast	y=0.13+0.007x+0.01x²	0.34	0.01	↑	y=7.2+0.12x	0.79	<0.01	↑	y=23.11+0.713x	0.76	<0.001	↑
Southeast	y=0.10+0.005x+0.01x²	0.40	0.01	↓↑	y=5.7-0.08x	0.77	<0.01	↓	y=18.4-0.412x	0.80	<0.01	↓
South	y=0.18-0.0007x-0.0003x³	0.02	0.84	─	y=7.7-0.18x	0.77	<0.01	↓	y=19.2-0.33x	0.59	<0.01	↓
Midwest	y=0.146+0.003x	0.03	0.44	─	y=7.7-0.06x	0.21	0.03	↓	y=30.7-0.30x-0.02x³	0.18	0.05	─

*, Trend

**Figure 2:**
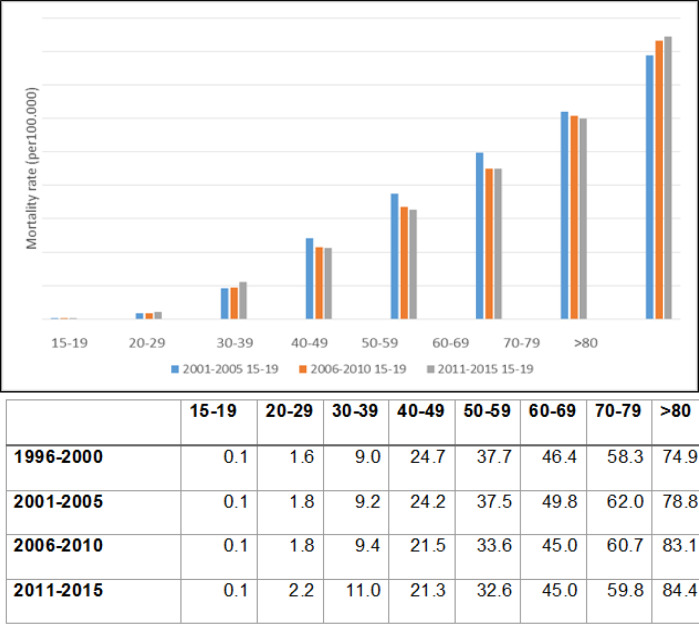
Mortality from Cervical Cancer in Brazilian Women in Different Age Groups in Four Periods (1996-2000, 2001-2005 e 2011-2015)

**Figure 3:**
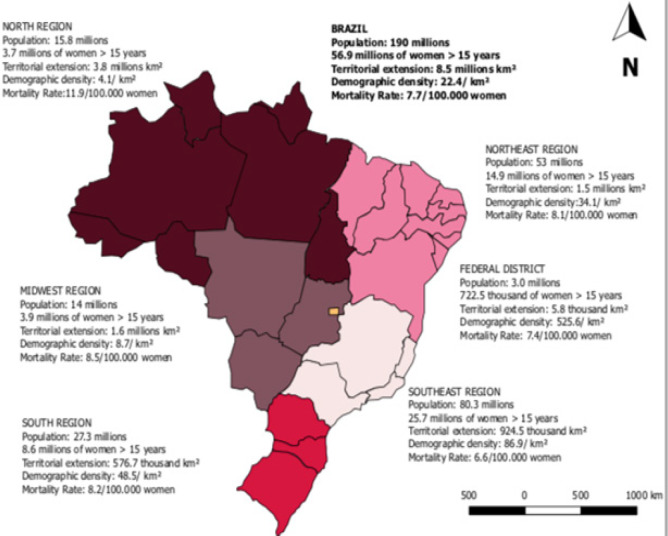
Distribution of Cervical Cancer Mortality Rates in the Brazilian States in the Period of 1996-2015

## Discussion

Studies of the distribution of CC mortality through temporal trends in women not included in screening programs, as assessed through regional and state disparities in Brazil, are still scarce, although they are essential for understanding the disease. This type of analysis provides important information for the development of cancer research and intervention programs (Fitzmauric et al., 2013), reinforces the potential for early diagnosis and appropriate treatment by the public health system (Unger-Saldaña, 2014), and enables extrapolation to similar countries because of the continental dimensions of Brazil.

This study showed an increasing trend in CC mortality in Brazilian women, especially among young and old women, revealing variations among the five regions of the country and among the 26 Brazilian states and the Federal District. The rates were higher in the last five years, indicating an increase in mortality in the country, mainly in the North and Northeast regions. The mortality rate among women aged 15-24 years showed an upward trend, while the rate among women aged 25-64 years remained constant; in women aged 65 years or older, the rates were also constant, but very high.

This result may be intrinsically related to the available guidelines because the predominant screening pattern in Brazil for women under 25 years and over 64 years is opportunistic, that is, they undergo a Pap smear when seeking care for other reasons. Therefore, only 20 to 25% of Pap smears fall outside the recommended age group (Inca, 2016).

The analysis of CC mortality, aiming to provide helpful information for the development of cancer research and intervention programs, has been a subject of debate in recent decades (Song et al., 2017; Vicus, 2014). This study provides results similar to those by the Ministry of Health: the North region (Inca et al., 2018) has the highest rates when standardized by the world population, followed by the Northeast (5.81/100 thousand) (Brasil, 2016) and the Midwest (18.32/100 thousand) regions (Inca et al., 2018). In contrast, a study carried out in Brazil showed a decrease in CC mortality in women from the Southeast and South regions, the most developed in the country (Girianelli et al., 2014) and the regions with the fourth highest mortality rates (Inca, 2018).

In other countries, such as China, CC incidence increased in 2013, compared to 2003 to 2007 (Song et al., 2017). In contrast, the CC mortality rate decreased by 0.9 in Poland (Leśniczak et al., 2015).

The increasing trend in CC mortality can be explained by a number of factors, including aging of the national population, unhealthy lifestyles and poorly structured public healthcare programs (Bode et al., 2015). In addition, the variety of information and inconsistencies regarding the sexual behaviors considered unsafe may lead to increased HPV infection (Bennetts et al., 2015). Recent assessments of CC incidence and mortality trends in high-, middle- and low-income countries have identified that mortality rates are declining in high-income countries and increasing in low- and middle-income countries (Rocha et al., 2017; Torre et al., 2015). Another factor that may be associated with the high incidence and prevalence of CC is lack of knowledge about HPV, screening programs and vaccine availability, as shown in a study in Asia (Akhtar et al., 2015). In Africa, most women with CC have advanced disease (Maranga et al., 2013), which may be related to personal factors, poor access to healthcare and poor knowledge of CC (Ni et al., 2014) as well as to the low quality of Pap smears, as demonstrated by the rate of unsatisfactory smears (Monirath et al., 2016).

Corroborating the results obtained herein, studies show that older women are at higher risk of cancer and but have the lowest rates of participation in CC screening programs (Cadete et al., 2017). The incidence in Denmark is bimodal, with peaks at 35-40 years and 75 years. However, screening is not offered for Danish women aged ≥ 65 years; however, screening women over 65 years of age can reduce CC incidence and mortality and appears to be cost-effective (Martelo et al., 2015). As in Denmark, Brazil has not yet implemented a population-based CC screening program that attracts women of all age groups. In Australia, the National Cervical Screening Program recommends screening with conventional cytology, every two years, in sexually active asymptomatic women between the ages of 18-20 and 69 years (Australian Government Department of Health, 2017).

In addition to these results, a study in Brazil showed that the age of first sexual intercourse among adolescents younger than 17 years old was a risk factor for high grade squamous intraepithelial lesions, as was a period of five years or more since first sexual intercourse, according to the natural history of the disease (Xavier-Junior, 2017). Another study suggests that biological prematurity and hormonal influence is two possible explanations for the association between early age of first sexual intercourse and higher CC risk (Ribeiro et al., 2015). All of these results indicate that the screening of sexually active women younger than 25 years may be useful because the rates of cervical intraepithelial neoplasia 3 (CIN3) are not negligible (Saslow et al., 2012).

The results from this study suggest that organized screening programs are working because the mortality rate in women in the age group targeted by the programs is constant in most Brazilian states, to the detriment of women who are not part of this population. Therefore, there is a need to extend the screening to women in the age groups that are not currently covered because there is a relationship between early age of first sexual intercourse and the natural history of the disease. Thus, health professionals need to know that young women need follow-up (Xavier-Junior, 2017).Our study has some limitations. The research was carried out by consulting a database, and it is possible that information is incorrectly compiled. However, the analysis of CC mortality trends in a country with large dimensions is essential for assessing the existing variation in mortality rates throughout the country. It should also be noted that secondary data may be a source of sensitive data and are effective for ecological studies in the country as the sole source of available data on mortality. In this sense, the study of mortality data is important because it allows determining the magnitude of the disease, in addition to serving as a tool to identify gaps in patient access to health services and potential advances in treatment (Albrecht et al., 2013).

CC mortality in women showed a growing trend in Brazil, especially in the North and Northeast regions of Brazil. The North region had the highest rates for all age groups. Mortality in younger women (15 to 24 years) is becoming more frequent, and in turn, a high rate of women aged 65 years and older are also being affected by this form of cancer. Thus, it is necessary to reconsider the public health policies that establish the age recommended for CC screening for each region. It is also necessary to consider the frequent changes that occur in personal habits such as sexual activity at early ages and at older ages, education level, marital status, cultural development, and aspects related to access to diagnosis, treatment and strategic local planning, especially for early detection among younger women. Thus, by considering the particularities of each region, the CC mortality rates may decrease in the country.

## References

[B1] Abu-Rmeileh NME, Gianicolo EAL, Bruni A (2016). Cancer mortality in the West Bank, Occupied Palestinian Territory. BMC Public Health.

[B2] Akhtar N, Saeed A, Urooj F (2015). Update knowledgeon cervical cancer incidence and prevalence in Asia. Epidemiol Etiol Diagn Treat Cervical Cancer.

[B3] Albrecht CAM, Amorim MHC, ZandonadeI E, Viana K, Calheiros JO (2013). Mortalidade por câncer de mama em hospital de referência em oncologia, Vitória, ES. Rev Bras Epidemiol.

[B4] Arab M, Noghabaei G (2014). Comparison of age standard incidence rate trends of gynecologic and breast cancer in Iran and other countries. Iranian J Publ Health.

[B6] Bennetts WM, Patel H, Welner S, de Sanjose S, Weiss TW (2015). Global availability of data on HPV genotype-distribution in cervical, vulvar and vaginal disease and genotype-specific prevalence and incidence of HPV infection in females. Infect Agent Cancer.

[B7] Bode AM, Dong Z, Wang H (2016). Cancer prevention and control: alarming challenges in China. Nat Sci Rev.

[B9] Cadete TJ, Burke SL, Stewart K, Howart T, Schonberg M (2017). Cultural and emotional determinants of cervical cancer screening among older Hispanic women. Health Care Women.

[B10] Campbell FN, Lara-Torre E (2009). Follow-up compliance of adolescents with cervical dysplasia in aninner-citypopulation. J Pediatr Adolesc Gynecol.

[B11] Castañón A, Landy R, Cuzick J, Sasieni P (2014). Cervical screening at age 50-64 years and the risk of cervical cancer at age 65 years and older: population-based case control study. PLoS Med.

[B12] Castañón A, Leung VM, Landy R, Lim AW, Sasieni P (2013). Characteristics and screening history of women diagnosed with cervical cancer aged 20-29 years. Br J Cancer.

[B13] Girianelli VR, Gamarra CJ, Silva GA (2014). Disparities in cervical and breast cancer mortality in Brazil. Rev Saude Publica.

[B15] Hammer A, Fuglsang K, Hogsbjerg K, Blaakaer J (2015). Screening for cervical cancer in women older than 65 years will probably reduce the incidence and mortality. Ugeskr Laeger.

[B20] Landy R, Birke H, Castanon A, Sasieni P 2014), Benefits and harms of cervical screening from age 20 years compared with screening from age 25 years. Br J Cancer.

[B21] Lowy DR, Schiller JT (2006). Prophylactic human papilloma virus vaccines. J Clin Invest.

[B22] Madeiro A, Rufino AC, Brandão NS, Santos IS (2016). Cervical cancer mortality trends in Piauí, 2000-2011. Cad Saude Colet.

[B23] Maeda TC (2012). Conhecimento de mulheres idosas sobre o exame de papanicolaou. Ciência Cuidado e Saúde.

[B24] Maranga IO, Hampson L, Oliver AW (2013). Analysis of factors contributing to the low survival of cervical cancer patients undergoing radiotherapy in Kenya. PLoS One.

[B25] Monirath H, Sokha E, Nicole H (2016). Prevalence of abnormal cervical cytology in HIV-negative women participating in a cervical cancer screening program in Calmette Hospital, Cambodia. Asian Pac J Cancer Prev.

[B27] Ni E, Mupepi SC, Siakwa MP, Sampselle CM (2014). Knowledge, practice, and barriers toward cervical cancer screening in Elmina, Southern Ghana. Int J Womens Health.

[B29] Ribeiro AA, Costa MC, Alves RR (2015). HPV infection and cervical neoplasia: associated risk factors. Infect Agent Cancer.

[B30] Rocha TA, Silva NC, Thomaz EB (2017). Primary health care and cervical cancer mortality rates in Brazil: A Longitudinal Ecological Study. JACM.

[B32] Snijders PJ, Steenbergen RD, Heideman DA, Meijer CJ (2006). HPV-mediated cervical carcinogenesis: concepts and clinical implications. J Pathol.

[B33] Song B, Ding C, Chen W (2017). Incidence and mortality of cervical cancer in China, 2013. Chin J Cancer Res.

[B34] Sousa AMV, Teixeira CCA, Medeiros SS (2016). Mortalidade por câncer do colo do útero no estado do Rio Grande do Norte, no período de 1996 a 2010: tendência temporal e projeções até 2030. Epidemiol Serv Saude.

[B35] Stormo AR de, de Moura L, Saraiya M (2014). Conhecimentos, atitudes e práticas relacionadas ao câncer de colo uterino de profissionais de saúde que atuam na rede de unidades básicas de saúde do Brasil. Oncologista.

[B36] (2017). Strobe. The Strengthening the Reporting of Observational Studies in Epidemiology Statement: guidelines for reporting observational studies. http://www.equator-network.org/reporting-guidelines/strobe/.

[B37] Torre LA, Siegel RL, Ward EM, Jemal A (2016). Global cancer incidence and mortality rates and trends an update. Epidemiol Cancer Biomarkers Prev.

[B38] Unger-Saldaña K (2014). Challenges to the early diagnosis and treatment of breast cancer in developing countries. World J Clin Oncol.

[B39] Vicus D, Sutradhar R, Lu Y (2014). The association between cervical cancer screening and mortality from cervical cancer: A population based case–control study. Ginecol Oncol.

[B40] Xavier-Júnior JCC, Vale DB, Vieira LF (2015). Results of screening for cervical cancer among pregnant and non-pregnant women in Brazil. Int J Gynaecol Obstet.

[B41] World Health Organization (2016). ICO Information Centre onHumanPapillomaVirus (HPV) and Cervical Cancer: Human papillomavirus and related cancers in Brazil.

[B42] World Health Organization (2019). Global. Human papillomavirus (HPV) and cervical câncer. Key facts. 2019..

